# Antibiotic therapy for small intestinal bacterial overgrowth: a systematic review of comparative efficacy, clinical–test discordance, and pediatric evidence gaps

**DOI:** 10.3389/fphar.2026.1908516

**Published:** 2026-09-10

**Authors:** Ana Maria Koller, Maria Oana Săsăran, Ancuța Lupu, Cristina Oana Mărginean

**Affiliations:** 1 Doctoral School of Medicine and Pharmacy, “George Emil Palade” University of Medicine, Pharmacy, Science, and Technology of Târgu Mureş, Targu Mures, Romania; 2 Department of Pediatrics 3, “George Emil Palade” University of Medicine, Pharmacy, Science, and Technology of Târgu Mureș, Targu Mures, Romania; 3 Department of Pediatrics, “Grigore T. Popa” University of Medicine and Pharmacy, Iaşi, Romania; 4 Department of Pediatrics 1, “George Emil Palade” University of Medicine, Pharmacy, Science and Technology of Târgu Mureș, Targu Mures, Romania

**Keywords:** antibiotic therapy, breath-test normalization, clinical response, intestinal methanogen overgrowth, metronidazole, pediatric treatment, rifaximin, small intestinal bacterial overgrowth

## Abstract

**Background:**

Small intestinal bacterial overgrowth (SIBO) and intestinal methanogen overgrowth (IMO) are heterogeneous conditions with poorly standardized antibiotic treatment. This review assessed clinical and test-based outcomes of SIBO therapy, phenotype-specific response, recurrence, and pediatric evidence on the same subject.

**Methods:**

PubMed/MEDLINE, Scopus, and Web of Science Core Collection were searched for English-language human studies published between January 2000 and April 2026. Randomized, non-randomized interventional, and observational studies evaluating antibiotic-based treatment for SIBO or methane-positive/IMO phenotypes were included. Owing to substantial heterogeneity, findings were synthesized narratively.

**Results:**

Thirty-nine studies were included. Rifaximin was the most frequently studied antibiotic, although outcomes varied considerably. In the only direct three-antibiotic comparison, metronidazole achieved the highest breath-test normalization, whereas rifaximin provided greater improvement in abdominal pain and bloating and fewer adverse events. Rifaximin plus neomycin showed the strongest comparative signal in methane-positive/IMO phenotypes. Clinical and test-based outcomes were frequently discordant, and pediatric evidence remained sparse and inconsistent.

**Conclusion:**

No antibiotic was universally superior. In adults, rifaximin appears to offer the most favorable overall balance between symptom improvement and tolerability, whereas metronidazole may achieve higher breath-test normalization in selected patients. Rifaximin plus neomycin showed the strongest comparative signal in methane-positive/IMO phenotypes. Evidence is insufficient to identify a preferred pediatric regimen. Treatment success should integrate symptoms, objective response, tolerability, recurrence, and patient phenotype.

## 1 Introduction

Small intestinal bacterial overgrowth (SIBO) has gained attention as a potential contributor to nonspecific gastrointestinal symptoms, but its diagnosis and treatment remain difficult to standardize. Patients commonly report bloating, abdominal pain, diarrhea, or excessive gas, symptoms that also occur in several functional and malabsorptive disorders (Koller et al., 2025a; Sieczkowska et al., 2016; Peña-Vélez et al., 2019; Collins and Lin, 2011; Berger et al., 2007). Increasing attention has been given to the role of the gut microbiota in gastrointestinal symptom generation and host intestinal homeostasis (Koller et al., 2025a; Sieczkowska et al., 2016; Quigley, 2015; Koller et al., 2025b).

SIBO is traditionally defined by an excessive bacterial burden in the small intestine, often by a proliferation over a threshold of 10^5^ colony-forming units per milliliter (Quigley and Quera, 2006). In practice, however, this definition is difficult to apply consistently. Culture-based thresholds vary across studies, and breath testing remains incompletely standardized, which complicates both diagnosis and assessment of treatment response (Sachdev and Pimentel, 2013).

The pathogenesis of SIBO is multifactorial. Impaired intestinal motility, altered host defense mechanisms, anatomical abnormalities, ileocecal valve dysfunction, delayed small-bowel transit, and reduced luminal acidity may all favor microbial stasis and overgrowth (Sieczkowska et al., 2016; Peña-Vélez et al., 2019; Chander et al., 2017). Clinical manifestations range from asymptomatic disease to persistent gastrointestinal symptoms and, in selected patients, lead to nutritional consequences (Sieczkowska et al., 2016; Bures et al., 2010; Korterink et al., 2015; Santos et al., 2020; Mello et al., 2018; Dos Reis et al., 2007; Saad and Chey, 2014). Children with short bowel syndrome appear particularly vulnerable, especially after loss of the ileocecal valve, where bacterial overgrowth may aggravate malabsorption, diarrhea, abdominal distension, growth failure and other complications (Goulet and Ruemmele, 2006; Jimenez et al., 2018; Velim et al., 2025). Nutritional status may also differ according to breath-test subtype, with recent adult data linking hydrogen/methane patterns to differences in vitamin D, ferritin, folate, fiber intake, and lactose intake (Wielgosz-Grochowska et al., 2024).

Diagnosis is further complicated by the overlap between SIBO, functional gastrointestinal disorders, and carbohydrate malabsorption. Lactose and fructose malabsorption, for example, may produce bloating, abdominal pain, diarrhea, and flatulence, symptoms that can mimic or coexist with SIBO (Simr et al., 2006; Goebel-Stengel et al., 2014). Similar diagnostic uncertainty exists in patients fulfilling criteria for irritable bowel syndrome (IBS), in whom breath-test abnormalities and antibiotic-associated symptom improvement have been reported, but are not always easy to interpret (Esposito et al., 2007; Morales-Guzmán et al., 2025; Pimentel et al., 2000). This overlap is biologically plausible, as molecular profiling studies have shown that fecal microbiota composition in IBS differs from that of healthy controls and may vary across symptom-based IBS subtypes (Kassinen et al., 2007). In children, this overlap may be even more complex, as disaccharidase deficiencies, secondary mucosal dysfunction, as well as suspected SIBO may produce similar clinical patterns (Hoskins et al., 2026).

Antibiotics remain the most commonly used treatment for SIBO, with rifaximin being the most frequently studied agent; metronidazole, norfloxacin, and trimethoprim–sulfamethoxazole have also been used in specific settings (Collins and Lin, 2011; Tahan et al., 2013; Kang et al., 2017; Ghoshal et al., 2016). The interest in rifaximin is also supported by IBS literature, a condition in which this minimally absorbed antibiotic has been associated with improvement in global symptoms and bloating, although these effects cannot be directly equated with SIBO eradication (Foxx-Orenstein, 2016). In pediatric patients, treatment decisions require particular caution because dosing, nutritional status, comorbidities, and pharmacokinetic considerations may influence both efficacy and safety (Standing et al., 2018). Across studies, however, antibiotic outcomes vary considerably. Symptom improvement does not always coincide with breath-test normalization, raising the question of whether treatment success should be defined primarily by eradication or by clinically meaningful improvement (Ghoshal et al., 2016; García-Collinot et al., 2020; Redo et al., 2024). A similar issue has been recognized in IBS antibiotic research, where patient-reported benefit, safety, duration of response, and recurrence are considered clinically relevant outcomes, rather than the reliance on a single physiological marker (Ozair et al., 2021).

This uncertainty is partly related to limitations of breath testing. Hydrogen and methane breath tests are non-invasive and widely used, but their interpretation depends on substrate choice, intestinal transit, patient preparation, methane production, and the presence of non-hydrogen-producing flora (Simr et al., 2006; Malik et al., 2011; Martyniak et al., 2025). These factors may lead to false-positive or false-negative results and may obscure the relationship between microbial changes and symptom response. In clinical cohorts, positive hydrogen or methane tests may also reflect patient context; for example, post-surgical patients may test positive for SIBO because of altered transit, whereas patients labeled as IBS may be less likely to have a positive test (Essa et al., 2021).

Methane-positive breath-test findings should be distinguished from bacterial overgrowth. Methane is produced by methanogenic archaea, which may proliferate in both the small intestine and the colon; accordingly, current consensus and position statements use the term intestinal methanogen overgrowth (IMO) rather than SIBO for methane-predominant phenotypes (Ghoshal et al., 2022; Silva et al., 2025). Several treatment studies, however, enrolled patients on the basis of methane-positive breath tests before the current terminology was adopted or without using the term IMO (Low et al., 2010; Pimentel et al., 2014). These studies were retained because they form part of the antibiotic-treatment evidence base, but methane-related populations and outcomes were interpreted separately whenever the reported data permitted.

Emerging evidence also suggests that treatment response may depend on baseline gas patterns, microbial profiles, underlying disease, and the broader therapeutic context, including probiotic regimens, dietary intervention, prokinetics, or multimodal approaches (García-Collinot et al., 2020; Redo et al., 2024; Pimentel et al., 2014; Rezaie et al., 2025; Li et al., 2020; Liu et al., 2022). In the same direction, pilot data in functional dyspepsia with SIBO suggest that non-antibiotic microbiome-modulating approaches, such as ursodeoxycholic acid, may influence symptoms and methane production, although these findings remain preliminary (Kim et al., 2020). Together, these observations challenge an exclusively eradication-centered view of SIBO therapy and support the need to evaluate antibiotic treatment using both clinical and microbiological outcomes.

Accordingly, this systematic review aims to evaluate antibiotic-based therapies for SIBO and methane-positive/IMO phenotypes reported within the SIBO treatment literature, with particular attention to clinical response, symptom improvement, breath-test normalization or microbiological eradication, recurrence, and variation across therapeutic strategies and clinical contexts.

## 2 Materials and methods

### 2.1 Study design and review question

This systematic review was conducted in accordance with the Preferred Reporting Items for Systematic Reviews and Meta-Analyses (PRISMA 2020) statement.

The review question followed the PICO framework. The population included pediatric and adult patients diagnosed with SIBO, as well as methane-positive/IMO phenotypes when reported within the SIBO treatment literature. The intervention focused on antibiotic-based treatments, either as monotherapy or in combination regimens. Comparators varied across studies and included placebo, lack of treatment, alternative antibiotic regimens, or adjunctive therapeutic strategies. The main outcomes assessed were clinical response, symptom improvement, breath-test normalization, microbiological eradication, remission, recurrence, relapse, and other treatment-related outcomes.

### 2.2 Search strategy

A comprehensive systematic literature search was conducted in PubMed/MEDLINE, Scopus, and Web of Science Core Collection to identify English-language human studies evaluating antibiotic-based therapy for small intestinal bacterial overgrowth. The search covered studies published between 1 January 2000 and 2 April 2026.

The search strategy combined terms related to small intestinal bacterial overgrowth, antibiotic or antimicrobial therapy, and treatment-related outcomes, including symptom response, clinical response, eradication, breath-test normalization, remission, recurrence, relapse, and other treatment-related outcomes. The search also incorporated terminology related to methane-positive phenotypes and intestinal methanogen overgrowth, including “intestinal methanogen overgrowth”, “IMO”, “methane-positive SIBO”, and related methane-predominant terms, in order to capture studies relevant to methane-positive/IMO phenotypes reported within the SIBO treatment literature.

All retrieved records were exported, merged, and deduplicated before screening. Potentially overlapping records were additionally checked manually. Reference lists of eligible studies and relevant reviews were manually screened to identify further potentially relevant articles. Database-specific search strategies and eligibility criteria are presented in Table 1.

**TABLE 1:** Search strategy and eligibility criteria.

Component	Description
Review focus	Antibiotic-based treatment strategies for small intestinal bacterial overgrowth (SIBO) and methane-positive/intestinal methanogen overgrowth (IMO) phenotypes reported within the SIBO treatment literature, with emphasis on clinical response, symptom improvement, breath-test normalization, microbiological eradication, recurrence, and other clinically relevant treatment outcomes in pediatric and adult populations.
Databases searched	PubMed/MEDLINE, Scopus, and Web of Science Core Collection.
Search timeframe	1 January 2000 to 2 April 2026.
Language restrictions	English.
Study designs included	Randomized controlled trials, non-randomized interventional studies, prospective and retrospective observational studies, and comparative clinical studies.
Database-specific strategy	PubMed/MEDLINE: (“small intestinal bacterial overgrowth” [Title/Abstract] OR SIBO [Title/Abstract] OR “intestinal methanogen overgrowth” [Title/Abstract] OR IMO [Title/Abstract] OR “methane-positive” [Title/Abstract] OR “methane positive” [Title/Abstract]) AND (antibiotic*[Title/Abstract] OR antimicrobial*[Title/Abstract] OR rifaximin [Title/Abstract] OR neomycin [Title/Abstract] OR metronidazole [Title/Abstract] OR norfloxacin [Title/Abstract] OR ciprofloxacin [Title/Abstract] OR amoxicillin [Title/Abstract] OR “trimethoprim-sulfamethoxazole” [Title/Abstract] OR “trimethoprim sulfamethoxazole” [Title/Abstract] OR cotrimoxazole [Title/Abstract] OR “rifamycin SV” [Title/Abstract]) AND (treatment [Title/Abstract] OR therapy [Title/Abstract] OR eradication [Title/Abstract] OR normalization [Title/Abstract] OR remission [Title/Abstract] OR recurrence [Title/Abstract] OR relapse [Title/Abstract] OR “clinical response” [Title/Abstract] OR “symptom improvement” [Title/Abstract] OR “breath test” [Title/Abstract]); filters: English language, human studies, and publication date 1 January 2000 to 2 April 2026. Scopus, main search: (“small intestinal bacterial overgrowth” OR SIBO) AND (antibiotic* OR antimicrobial* OR rifaximin OR neomycin OR metronidazole OR norfloxacin OR ciprofloxacin OR amoxicillin OR “trimethoprim-sulfamethoxazole” OR “trimethoprim sulfamethoxazole” OR cotrimoxazole OR “rifamycin SV”) AND (treatment OR therapy OR eradication OR normalization OR remission OR recurrence OR relapse OR “clinical response” OR “symptom improvement” OR “breath test”). Scopus, targeted methane/IMO search: (“intestinal methanogen overgrowth” OR “methane-positive SIBO” OR “methane positive SIBO” OR “methane-producing SIBO” OR “methane producing SIBO” OR “methane-predominant SIBO” OR “methane predominant SIBO”) AND (rifaximin OR neomycin OR antibiotic* OR antimicrobial* OR treatment OR therapy). Both Scopus searches were conducted within article titles, abstracts, and keywords; filters were publication years 2000–2026, English language, subject area Medicine, and document type Article. Records published after 2 April 2026 were manually excluded. Web of Science Core Collection, main search: (“small intestinal bacterial overgrowth” OR SIBO) AND (antibiotic OR antibiotics OR antimicrobial OR rifaximin OR neomycin OR metronidazole OR norfloxacin OR ciprofloxacin OR cotrimoxazole OR rifamycin); filters: publication years 2000–2026, English language, and document type Article. Web of Science Core Collection, targeted methane/IMO search: (“intestinal methanogen overgrowth” OR “methane-positive SIBO” OR “methane positive SIBO” OR “methane-producing SIBO” OR “methane producing SIBO” OR “methane-predominant SIBO” OR “methane predominant SIBO”) AND (rifaximin OR neomycin OR antibiotic OR antibiotics OR antimicrobial OR treatment OR therapy); filters: publication years 2000–2026, English language, and document type Article. Records published after 2 April 2026 were manually excluded.
Search strategy rationale	The search strategy was designed to identify studies evaluating antibiotic-based treatment across different SIBO populations, diagnostic approaches, antibiotic regimens, and clinical or microbiological outcomes, without restricting retrieval to a specific associated disorder or diagnostic modality. Methane-related and IMO terminology was incorporated because methane-positive phenotypes have historically been reported within the SIBO literature, although they are conceptually distinct from bacterial overgrowth and were interpreted separately where study-level data permitted. Terms referring to adjunctive therapies were not made mandatory, thereby reducing the likelihood of omitting studies in which antibiotics represented the principal therapeutic component of a combination or multimodal regimen.
Additional sources	Reference lists of eligible studies and relevant systematic reviews were manually screened to identify additional potentially relevant reports.
Inclusion criteria	Full-text original articles published in English between 1 January 2000 and 2 April 2026; pediatric and/or adult populations; randomized controlled trials, non-randomized interventional studies, prospective or retrospective observational studies, or comparative clinical studies; patients diagnosed with SIBO or methane-positive/IMO phenotypes according to study-defined criteria; antibiotic-based treatment administered as monotherapy, comparative therapy, retreatment, or as part of a combination or multimodal regimen; and reporting of at least one relevant outcome, including clinical response, symptom improvement, breath-test normalization, microbiological eradication, remission, recurrence, relapse, adverse events, or another treatment-related outcome.
Exclusion criteria	Animal or in vitro studies; articles outside the predefined publication period; reviews, systematic reviews, meta-analyses, editorials, letters, expert opinions, and narrative reviews; conference abstracts without accessible full text; case reports or very small case series; study protocols without outcome data; studies not primarily evaluating antibiotic-based treatment in SIBO or methane-positive/IMO populations; and studies focused exclusively on prevalence, diagnostic accuracy, pathophysiology, microbiome characterization, or mechanistic outcomes without relevant clinical or eradication-related treatment data. Reviews, meta-analyses, consensus documents, and guidelines were excluded from the qualitative synthesis but were used to contextualize the findings in the Introduction and Discussion.
Screening process	Titles and abstracts were screened independently, followed by full-text assessment of potentially eligible reports. Disagreements regarding eligibility were resolved through discussion and consensus.
Data extraction	Data were extracted using a standardized form capturing study design, population characteristics, diagnostic method and criteria, SIBO or methane/IMO phenotype, antibiotic regimen, comparator, follow-up duration, microbiological outcomes, clinical outcomes, adverse events, recurrence data, and relevant methodological limitations.

### 2.3 Eligibility criteria and study selection

Studies were considered eligible if they included pediatric and/or adult patients diagnosed with SIBO, or methane-positive/IMO phenotypes when reported within the SIBO treatment literature, evaluated antibiotic-based treatment strategies, and reported at least one relevant treatment-related outcome, including clinical response, symptom improvement, breath-test normalization, eradication, remission, recurrence, relapse, adverse events, or other treatment-related outcomes.

**FIGURE 1:**
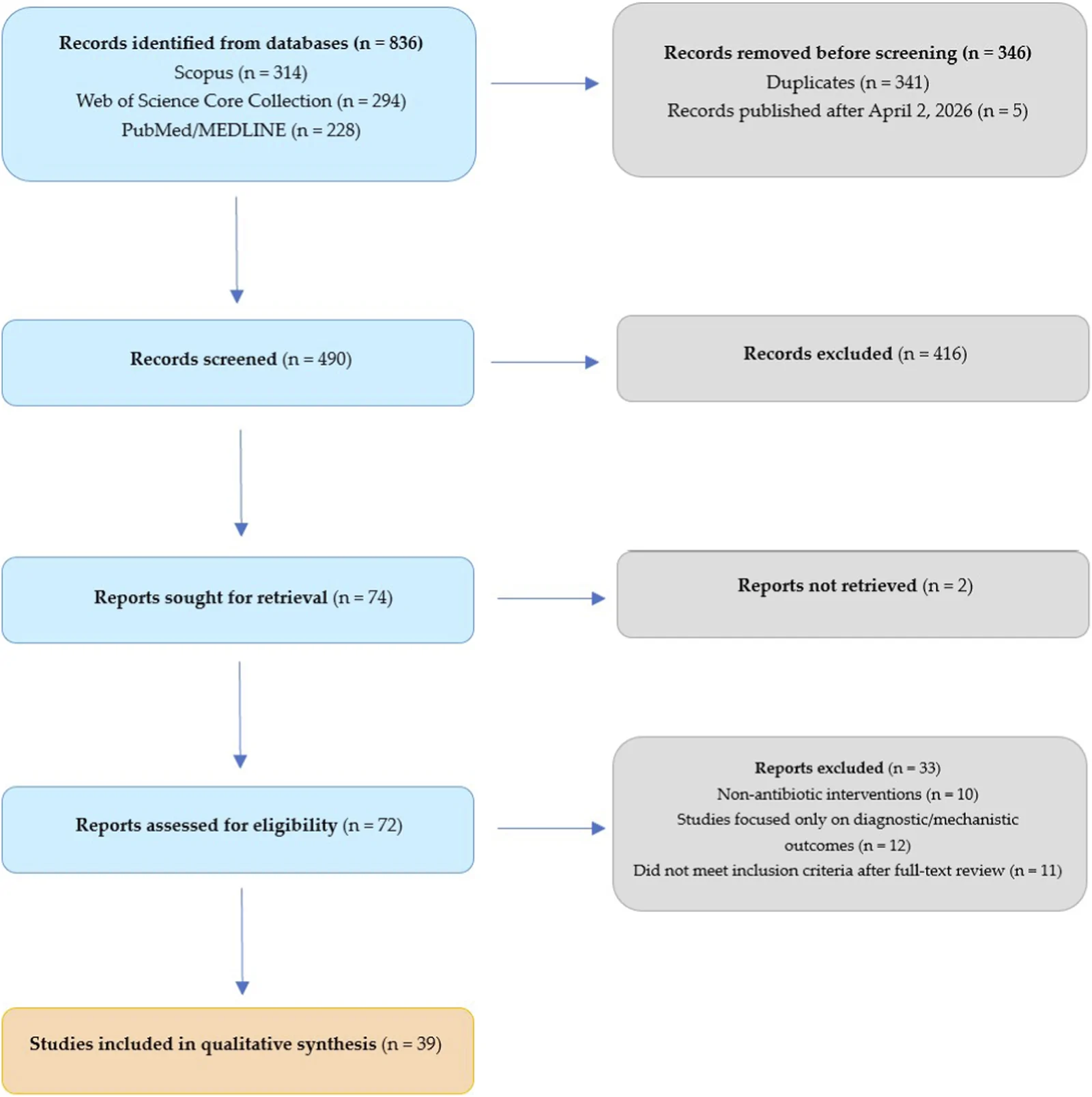
PRISMA 2020 flow diagram summarizing the study selection.

Titles and abstracts were screened independently, followed by full-text assessment of potentially eligible articles. Disagreements regarding study eligibility were resolved through discussion and consensus. The study selection process is presented in a PRISMA 2020 flow diagram (Figure 1).

### 2.4 Diagnostic definition of SIBO and methane-positive/IMO phenotypes

Because diagnostic approaches vary across studies, articles were considered eligible if SIBO had been diagnosed using a reported study-defined method, including glucose hydrogen breath testing, lactulose hydrogen breath testing, combined hydrogen/methane breath testing, or, where available, microbiological culture-based methods. Studies reporting methane-positive patients or IMO were retained when these phenotypes were described within the SIBO treatment literature. Methane-positive/IMO findings were extracted and interpreted separately where possible. Diagnostic criteria, gas thresholds, substrates, and follow-up definitions were recorded as reported in the original studies. Because methanogens are archaea rather than bacteria, methane-positive findings were classified as IMO rather than SIBO where study-level reporting permitted. Studies using the historical terminology “methane-positive SIBO” were retained but interpreted as methane-positive/IMO phenotypes.

### 2.5 Data extraction and risk of bias assessment

The risk of bias was assessed according to study design. Randomized trials were evaluated with the Cochrane Risk of Bias 2 (RoB 2) tool (Sterne et al., 2019), while non-randomized comparative, observational, and before–after interventional studies were assessed using the Risk Of Bias In Non-Randomized Studies of Interventions (ROBINS-I) tool (Sterne et al., 2016). For RoB 2, judgments were classified as “low risk of bias”, “some concerns”, or “high risk of bias”; for ROBINS-I, judgments were classified as “low”, “moderate”, “serious”, or “critical risk of bias”. Overall judgments were derived in accordance with the guidance provided for each instrument. Assessments were performed independently by the authors, and any disagreements were re-solved by discussion until consensus was reached. For non-randomized studies without a clearly concurrent comparator, ROBINS-I judgments were interpreted with caution in view of the inherent limitations of these designs. Overall risk-of-bias judgments are presented in Ta-ble 3.

### 2.6 Data synthesis

Given the heterogeneity in study design, patient populations, diagnostic methods, interventions, comparators, and reported outcomes, the findings were synthesized using a structured qualitative narrative approach. The synthesis focused on antibiotic efficacy, clinical response, the relationship between symptom improvement and breath-test normalization or microbiological eradication, combination and multimodal treatment strategies, recurrence and retreatment, determinants of treatment response, and differences between SIBO and methane-positive/IMO phenotypes.

Combination or multimodal treatment studies were retained when an antibiotic was a predefined component of the therapeutic regimen. When an antibiotic-only comparator was available, the incremental effect of the adjunctive intervention was considered. When the study design did not allow the antibiotic effect to be isolated, outcomes were attributed to the complete therapeutic regimen and were not interpreted as evidence of the independent efficacy of the antibiotic component.

## 3 Results

The study selection process is shown in Figure 1. The database searches identified 836 records. Following removal of duplicates and records published after the predefined cutoff date, 490 records underwent title and abstract screening. Seventy-four reports were sought for retrieval, of which two could not be obtained. Seventy-two full-text reports were assessed for eligibility, and 39 studies met the inclusion criteria for the qualitative synthesis. The included studies were heterogeneous in terms of design, population, diagnostic approach, SIBO or methane/IMO classification, antibiotic regimen, comparator, follow-up duration, and outcome reporting. Main study characteristics are summarized in Table 2, with detailed study-level data provided in Supplementary Table S1.

**TABLE 2:** Condensed overview of included studies evaluating antibiotic-based therapy for SIBO and methane-positive/IMO phenotypes.

Therapeutic focus	Study	Design	Population/context	Antibiotic-based strategy	Microbiological outcome	Clinical outcome
Rifaximin monotherapy and dose-ranging studies	(Di et al., 2000)	Double-blind RCT	Adults with SIBO	Rifaximin vs. chlortetracycline	Higher breath-test normalization with rifaximin	Greater symptom response with rifaximin
	(Lauritano et al., 2005)	Randomized dose-finding study	Adults with SIBO	Rifaximin dose comparison	Dose-dependent GBT normalization	Highest normalization at 1,200 mg/day; tolerability preserved
	(Majewski et al., 2007)	Prospective open-label study	Symptomatic adults with SIBO	Rifaximin monotherapy	GBT normalized in approximately half	Symptom response was more evident in diarrhea-predominant presentations
	(Scarpellini et al., 2007)	Prospective randomized study	Adults with SIBO	High-dose vs. standard-dose rifaximin	Higher normalization with 1,600 mg/day	Higher-dose regimen had similar tolerability
	(Tuteja et al., 2019)	Double-blind placebo-controlled RCT	Adults with IBS	Rifaximin vs. placebo	No significant breath-test benefit	No significant symptom benefit vs. placebo
	(Lee et al., 2019)	Retrospective observational study	Adults with IBS/SIBO	Rifaximin monotherapy	Not reported	Symptoms improved without meaningful weight or metabolic changes
	(Zhuang et al., 2020)	Prospective study	Adults with IBS-D	Rifaximin monotherapy	Partial LHBT normalization	GI symptoms and quality of life improved
	(Li et al., 2020)	Prospective mechanistic study	Adults with IBS-D	Rifaximin monotherapy	Eradication was not the primary endpoint	Response varied according to baseline microbiota composition
	(Chojnacki et al., 2022)	Before–after interventional study	Adults with SIBO-D/SIBO-C	Repeated rifaximin cycles	Eradication was not the primary endpoint	GI and psychological symptoms improved
	(Liu et al., 2022)	Clinical/mechanistic study	Adults with IBS-D stratified by breath-test status	Rifaximin	Eradication was not the primary endpoint	Symptom response was greater in SIBO-positive patients
	(García-Cedillo et al., 2025)	Prospective open-label pilot study	IBS without constipation, SIBO, and lactose intolerance	Rifaximin-alpha	Partial breath-test normalization	Symptoms, lactose maldigestion, and lactase-related measures improved
Comparative antibiotics, eradication-response, recurrence, and retreatment	(Pimentel et al., 2000)	Prospective database/open-label treatment study	IBS with abnormal LHBT suggestive of SIBO	Open-label antibiotics	Eradication was associated with symptom response	48% no longer met Rome criteria after successful eradication
	(Madrid and Defilippi)	Randomized controlled study	Adults with liver cirrhosis	Alternating norfloxacin/neomycin	Antibiotics reduced SIBO	Motility, OCTT, SIBO, and liver-function indices improved
	(Esposito et al., 2007)	Observational study	Adults initially diagnosed with IBS	Rifaximin; ciprofloxacin if positivity persisted	Approximately 59% became breath-test negative after rifaximin	Symptoms decreased significantly after rifaximin
	(Yang et al., 2008)	Retrospective chart review	IBS with abnormal LBT	Rifaximin vs. other antibiotics; retreatment assessed	LBT normalization predicted response	Rifaximin showed higher clinical response and better retreatment success
	(Lauritano et al., 2008)	Prospective follow-up study	Adults successfully decontaminated after SIBO therapy	Post-rifaximin follow-up	Recurrence increased over 9 months	Recurrence was associated with symptom relapse, older age, PPI use, and appendectomy
	(Ghoshal et al., 2016)	Double-blind placebo-controlled RCT	IBS stratified by SIBO status	Norfloxacin vs. placebo	Higher test normalization with norfloxacin	Symptom resolution was more frequent in SIBO-positive IBS patients
	(Melchior et al., 2017)	Pilot randomized trial	Adults with SIBO and gas-related symptoms	Metronidazole vs. simethicone/activated charcoal	Not applicable	Metronidazole reduced gas-related symptoms
	(Richard et al., 2021)	Retrospective comparative cohort	Adults with SIBO	Rotating vs. single-course antibiotics	Higher remission with rotating antibiotics	Rotating regimens were associated with better remission and QoL-related outcomes
	(Connor et al., 2025)	Open-label pilot randomized trial	Adults with SIBO	Rifamycin SV-MMX BID vs. TID	Breath-test response reported	Higher-frequency dosing produced greater symptom improvement
	(Von Muhlenbrock et al., 2025)	Prospective randomized double-blind comparative study	Adults with IBS and SIBO	Rifaximin vs. ciprofloxacin vs. metronidazole	Highest eradication with metronidazole	Rifaximin improved pain and bloating more and was associated with fewer adverse events
Combination, adjunctive, and multimodal approaches	(Low et al., 2010)	Retrospective chart review	Methane-positive IBS/IMO phenotype	Rifaximin, neomycin, or combination	Methane eradication was highest with combination therapy	Combination therapy produced the highest clinical response; interpreted as a methane-positive/IMO phenotype
	(Furnari et al., 2010)	Randomized clinical trial	Adults with SIBO and predisposing conditions	Rifaximin ± partially hydrolyzed guar gum	Eradication was higher with combination therapy	Clinical improvement was similar among patients with successful eradication
	(García-Collinot et al., 2020)	Open pilot randomized clinical trial	Systemic sclerosis with SIBO	Metronidazole ± Saccharomyces boulardii	Eradication was higher with combination therapy	Abdominal pain, bloating, and flatulence improved
	(Yu et al., 2021)	Comparative clinical study	Adults with IBS/SIBO	Antibiotics vs. microbiota-directed therapy	Not clearly reported	Symptoms improved together with changes in inflammatory markers
	(Kim et al., 2022)	Randomized blinded three-arm trial	Functional dyspepsia with SIBO	Rifaximin, mosapride, or combination	Eradication differed modestly	Gas-related symptoms improved more with rifaximin-based therapy
	(Jo et al., 2024)	Double-blind placebo-controlled RCT	Functional bloating with SIBO	Rifaximin plus trimebutine vs. rifaximin plus placebo	No added eradication benefit	Add-on trimebutine improved bloating and gas-related symptoms
	(Redo et al., 2024)	Prospective comparative interventional study	Adults with SIBO	Antibiotics plus diet ± adjunctive interventions	Gas normalization did not differ significantly	Multimodal treatment improved symptoms, especially in methane-predominant patients
Specific clinical populations	(Jahraus et al., 2007)	Retrospective case review	Patients with GI symptoms during abdominopelvic radiotherapy and suspected or breath-test-defined SIBO	Rifaximin 800–1,200 mg/day during radiotherapy and for 2–4 weeks afterward	Post-treatment breath-test normalization was not systematically assessed	Partial symptom relief was reported in 94% and global relief in 81% of evaluable patients
	(Parodi et al., 2008)	Prospective clinical study	Systemic sclerosis with SIBO	Rifaximin	SIBO eradication was frequent	Intestinal symptoms improved after eradication
​	(Franco et al., 2015)	Retrospective cohort study	Patients undergoing upper endoscopy with culture-defined SIBO	Rifaximin, ciprofloxacin, metronidazole, or other antibiotics	SIBO was defined by duodenal aspirate culture; post-treatment eradication was not systematically assessed	Among culture-positive patients, improvement was similar with and without antibiotics (53% vs. 46.5%)
	(Deng et al., 2016)	Prospective interventional study	Postoperative colorectal cancer patients with SIBO	Rifaximin	One-third converted to GHBT-negative status	Overall GI symptoms improved, especially diarrhea
	(Konrad et al., 2018)	Comparative non-randomized study	SIBO with H. pylori infection	Rifaximin plus amoxicillin	LHBT improvement reported	Symptoms improved in patients with SIBO/H. pylori co-infection
	(Furnari et al., 2019)	Randomized controlled study	Cystic fibrosis with SIBO	Rifaximin vs. no treatment	High eradication with rifaximin	GI symptoms and nutritional parameters improved
	(Chaudhry et al., 2025)	Retrospective clinical utility study	Gas-bloat symptoms after antireflux surgery	Antibiotics for SIBO-positive patients	Eradication was not the primary endpoint	Gas-bloat, GERD-HRQL, and patient satisfaction improved
	(Chidambaram et al., 2025)	Retrospective cohort study	Reflux/LPR symptoms with SIBO or IMO	Antibiotics for SIBO/IMO	Eradication was not the primary endpoint	GERD-HRQL and RSI improved; PPI use decreased
Pediatric or pediatric-relevant studies	(Collins and Lin, 2011)	Double-blind placebo-controlled RCT	Children with chronic abdominal pain	Rifaximin vs. placebo	Low LHBT normalization	No significant symptom benefit vs. placebo
	(Lisowska et al., 2011)	Interventional clinical study	Cystic fibrosis patients with SIBO	Oral or IV antibiotics	Eradication was not the primary endpoint	Fat digestion and absorption improved, especially with oral antibiotics
	(Scarpellini et al., 2013)	Preliminary prospective open-label study	Children with IBS and SIBO	Rifaximin	LHBT normalization in most treated children	Symptoms improved mainly after successful normalization

Abbreviations: BID, twice daily; GBT, glucose breath test; GERD-HRQL, Gastroesophageal Reflux Disease Health-Related Quality of Life questionnaire; GHBT, glucose hydrogen breath test; GI, gastrointestinal; IBS, irritable bowel syndrome; IBS-D, diarrhea-predominant irritable bowel syndrome; IMO, intestinal methanogen overgrowth; IV, intravenous; LBT, lactulose breath test; LHBT, lactulose hydrogen breath test; LPR, laryngopharyngeal reflux; MMX, multi-matrix system; OCTT, orocecal transit time; PPI, proton pump inhibitor; QoL, quality of life; RCT, randomized controlled trial; RSI, reflux symptom index; SIBO, small intestinal bacterial overgrowth; SIBO-C, constipation-predominant SIBO; SIBO-D, diarrhea-predominant SIBO; TID, three times daily.

### 3.1 Risk of bias assessment

The included studies differed considerably in methodological quality. Among randomized studies, most were classified as having some concerns or high risk of bias, mainly because of small sample sizes, incomplete reporting of randomization or allocation procedures, open-label or pilot designs, incomplete follow-up, and reliance on subjective symptom-based outcomes. Non-randomized studies were generally judged to have serious risk of bias, primarily because of confounding, selection bias, retrospective or uncontrolled designs, lack of concurrent comparator groups, and heterogeneity in outcome assessment. For this reason, treatment effects should be interpreted cautiously, particularly when symptom-based outcomes are compared with eradication or breath-test normalization across studies with different designs and diagnostic methods. Overall risk-of-bias judgments are presented in Table 3.

**TABLE 3:** Risk of biasc summary.

Study	Design	Tool	Overall risk of bias
RoB 2 tool			
(Di et al., 2000)	Double-blind randomized controlled trial	RoB 2	Some concerns
(Madrid and Defilippi)	Randomized controlled study	RoB 2	Some concerns
(Lauritano et al., 2005)	Prospective randomized dose-finding study	RoB 2	Some concerns
(Scarpellini et al., 2007)	Prospective randomized dose-comparison study	RoB 2	Some concerns
(Furnari et al., 2010)	Randomized clinical trial	RoB 2	Some concerns
(Collins and Lin, 2011)	Double-blind placebo-controlled RCT	RoB 2	Some concerns
(Ghoshal et al., 2016)	Double-blind placebo-controlled RCT	RoB 2	Some concerns
(Melchior et al., 2017)	Pilot randomized trial, unblinded	RoB 2	High risk of bias
(Furnari et al., 2019)	Open-label randomized controlled trial	RoB 2	High risk of bias
(Tuteja et al., 2019)	Double-blind placebo-controlled RCT	RoB 2	Some concerns
(García-Collinot et al., 2020)	Open pilot randomized clinical trial	RoB 2	High risk of bias
(Kim et al., 2022)	Randomized blinded three-arm trial	RoB 2	Some concerns
(Jo et al., 2024)	Double-blind placebo-controlled RCT	RoB 2	Low risk of bias
(Connor et al., 2025)	Open-label pilot randomized trial	RoB 2	High risk of bias
(Von Muhlenbrock et al., 2025)	Prospective randomized double-blind three-arm trial	RoB 2	Some concerns
ROBINS-I tool			
(Pimentel et al., 2000)	Prospective database/open-label treatment study	ROBINS-I	Serious risk of bias
(Esposito et al., 2007)	Observational study	ROBINS-I	Serious risk of bias
(Jahraus et al., 2007)	Retrospective case review	ROBINS-I	Serious risk of bias
(Majewski et al., 2007)	Prospective open-label single-arm study	ROBINS-I	Serious risk of bias
(Lauritano et al., 2008)	Prospective follow-up study after successful SIBO decontamination	ROBINS-I	Serious risk of bias
(Parodi et al., 2008)	Prospective clinical study in systemic sclerosis-associated SIBO	ROBINS-I	Serious risk of bias
(Yang et al., 2008)	Retrospective chart review	ROBINS-I	Serious risk of bias
(Low et al., 2010)	Retrospective chart review in methane-positive patients	ROBINS-I	Serious risk of bias
(Lisowska et al., 2011)	Interventional clinical study in cystic fibrosis patients with SIBO	ROBINS-I	Serious risk of bias
(Scarpellini et al., 2013)	Preliminary open-label prospective single-arm trial	ROBINS-I	Serious risk of bias
(Franco et al., 2015)	Retrospective cohort study	ROBINS-I	Serious risk of bias
(Deng et al., 2016)	Prospective interventional study	ROBINS-I	Serious risk of bias
(Konrad et al., 2018)	Comparative non-randomized study	ROBINS-I	Serious risk of bias
(Lee et al., 2019)	Retrospective observational study	ROBINS-I	Serious risk of bias
(Li et al., 2020)	Prospective mechanistic before–after study	ROBINS-I	Serious risk of bias
(Zhuang et al., 2020)	Prospective single-arm study	ROBINS-I	Serious risk of bias
(Richard et al., 2021)	Retrospective comparative cohort	ROBINS-I	Serious risk of bias
(Yu et al., 2021)	Comparative non-randomized clinical study	ROBINS-I	Serious risk of bias
(Chojnacki et al., 2022)	Non-randomized interventional before–after study	ROBINS-I	Serious risk of bias
(Liu et al., 2022)	Non-randomized clinical/mechanistic study	ROBINS-I	Serious risk of bias
(Redo et al., 2024)	Prospective comparative interventional study with center-based intervention groups	ROBINS-I	Serious risk of bias
(Chaudhry et al., 2025)	Retrospective clinical utility study	ROBINS-I	Serious risk of bias
(Chidambaram et al., 2025)	Retrospective cohort study	ROBINS-I	Serious risk of bias
(García-Cedillo et al., 2025)	Prospective open-label pilot study	ROBINS-I	Serious risk of bias

Abbreviations: RCT, randomized controlled trial; RoB 2, Cochrane Risk of Bias 2; ROBINS-I, Risk Of Bias in Non-randomized Studies of Interventions.

### 3.2 Efficacy of antibiotic therapies

Reported antibiotic efficacy varied according to patient population, treatment regimen, and outcome definition. In a placebo-controlled study of patients with IBS, rifaximin did not improve symptoms or breath-test outcomes compared with placebo (Tuteja et al., 2019). By contrast, in adults with cystic fibrosis and SIBO, rifaximin was associated with a higher eradication rate than no treatment (90% vs. 33.3%), together with improvement in selected gastrointestinal symptoms and nutritional parameters (Furnari et al., 2019). In functional gastrointestinal disorders with documented SIBO, other rifaximin-based studies reported breath-test normalization rates of approximately 30%–35%, whether rifaximin was administered alone or combined with a prokinetic agent. The addition of a prokinetic did not increase normalization rates, although gas-related symptoms, particularly bloating, improved in some patients (Kim et al., 2022; Jo et al., 2024).

In patients with IBS and confirmed SIBO, norfloxacin was associated with greater symptom resolution than placebo; more treated patients also no longer fulfilled Rome III criteria and converted to negative SIBO testing (Ghoshal et al., 2016). A prospective randomized double-blind study in Chilean adults with Rome IV-defined IBS and lactulose breath test-confirmed SIBO found the highest post-treatment breath-test normalization rate with metronidazole, whereas rifaximin produced greater improvement in abdominal pain and bloating and was associated with fewer adverse events than the comparator regimens (Von Muhlenbrock et al., 2025). In systemic sclerosis-associated SIBO, metronidazole plus *S. boulardii* (*Saccharomyces boulardii*) achieved a higher breath-test normalization rate than metronidazole alone (García-Collinot et al., 2020). In a comparative cohort study, rotating antibiotic regimens were associated with more frequent breath-test normalization than single-course regimens (Richard et al., 2021). In a randomized study, multimodal regimens combining antibiotics with dietary and adjunctive interventions produced greater clinical improvement, although normalization of breath-test gas values did not differ significantly between groups (Redo et al., 2024).

Retrospective studies also yielded contrasting clinical findings. Among patients undergoing abdominopelvic radiotherapy, 94% reported improvement in at least one gastrointestinal symptom and 81% reported improvement in all presenting symptoms after rifaximin treatment. By contrast, in a tertiary-center cohort with culture-confirmed SIBO, clinical improvement was similar in antibiotic-treated and untreated patients (53% vs. 46.5%) (Jahraus et al., 2007; Franco et al., 2015).

Pediatric findings were likewise inconsistent. In a double-blind placebo-controlled trial of children with chronic abdominal pain, rifaximin did not significantly improve symptoms compared with placebo, and the lactulose breath test normalized in only 20% of treated children (Collins and Lin, 2011). Conversely, in a preliminary study of children with IBS and abnormal lactulose hydrogen/methane breath testing, rifaximin normalized the lactulose breath test in 64% of SIBO-positive participants. Among children in whom the breath test normalized, abdominal pain, bloating, and flatulence also improved. Treatment adherence was high, and no clinically relevant adverse events were reported (Scarpellini et al., 2013).

The comparative efficacy and clinical impact of the main antibiotic strategies are summarized in Table 4.

**TABLE 4:** Selected studies reporting microbiological/test-based and clinical outcomes following antibiotic-based therapy for SIBO and methane-positive/IMO phenotypes.

Study	Study design	Antibiotic-based strategy	Microbiological/test-based outcome	Clinical outcome and interpretation
(Jahraus et al., 2007)	Retrospective case review	Rifaximin 800–1,200 mg/day during abdominopelvic radiotherapy and for 2–4 weeks afterward	Post-treatment breath-test normalization was not systematically assessed	Among 16 evaluable patients, 94% reported improvement in at least some presenting GI symptoms and 81% reported improvement in all presenting symptoms; findings were based on an uncontrolled retrospective assessment
(Collins and Lin, 2011)	Double-blind placebo-controlled RCT	Rifaximin vs. placebo	LHBT normalization: 20% vs. 14%	No significant symptom improvement was observed with rifaximin compared with placebo
(Scarpellini et al., 2013)	Preliminary prospective open-label study	Rifaximin	LHBT normalization: 64%	Abdominal pain, bloating, and flatulence improved mainly among children who achieved breath-test normalization
(Franco et al., 2015)	Retrospective cohort study	Antibiotic treatment, predominantly rifaximin; ciprofloxacin, metronidazole, or other antibiotics were also used	SIBO was confirmed by duodenal aspirate culture; post-treatment microbiological eradication was not systematically assessed	Among culture-positive patients, clinical improvement did not differ significantly between those treated and not treated with antibiotics (53.0% vs. 46.5%)
(Ghoshal et al., 2016)	Double-blind placebo-controlled RCT	Norfloxacin vs. placebo	Breath-test normalization: 50% vs. 0%	Symptom resolution was more frequent with norfloxacin in SIBO-positive patients with IBS
(Tuteja et al., 2019)	Double-blind placebo-controlled RCT	Rifaximin vs. placebo	No significant between-group difference in breath-test findings	Rifaximin did not produce a significant symptom benefit compared with placebo
(Furnari et al., 2019)	Open-label randomized controlled study	Rifaximin vs. no treatment	Breath-test normalization: 90% vs. 33.3%	Rifaximin was associated with improvement in selected GI symptoms and nutritional parameters
(García-Collinot et al., 2020)	Open pilot randomized clinical trial	Metronidazole vs. metronidazole plus Saccharomyces boulardii	Breath-test normalization: 25% vs. 55%	Combination treatment was associated with improvement in abdominal pain, bloating, and flatulence; findings represent the effect of the combined regimen
(Richard et al., 2021)	Retrospective comparative cohort	Rotating vs. single-course antibiotics	Remission: 70.0% vs. 50.8%	Rotating regimens were associated with higher remission rates and better bloating- and QoL-related outcomes
(Kim et al., 2022)	Randomized blinded three-arm trial	Rifaximin vs. mosapride vs. rifaximin plus mosapride	Breath-test normalization: 32.1%, 17.2%, and 34.6%, respectively	Gas-related symptoms improved more with rifaximin-based therapy, whereas normalization rates differed only modestly between groups
(Jo et al., 2024)	Double-blind placebo-controlled RCT	Rifaximin alone vs. rifaximin plus trimebutine	Breath-test normalization: 35.9% vs. 34.1%	Add-on trimebutine improved bloating and gas-related symptoms without increasing breath-test normalization
(Redo et al., 2024)	Prospective comparative interventional study	Antibiotics plus low-FODMAP diet with or without adjunctive interventions	Gas normalization did not differ significantly between groups	Multimodal treatment produced greater symptomatic improvement, particularly in methane-predominant patients; the independent antibiotic effect could not be isolated
(Von Muhlenbrock et al., 2025)	Prospective randomized double-blind three-arm trial	Rifaximin vs. ciprofloxacin vs. metronidazole	Breath-test normalization: 59%, approximately 54%, and 79%, respectively	Metronidazole produced the highest normalization rate, whereas rifaximin was associated with greater improvement in abdominal pain and bloating and fewer adverse effects

Abbreviations: GI, gastrointestinal; IBS, irritable bowel syndrome; IMO, intestinal methanogen overgrowth; LHBT, lactulose hydrogen breath test; QoL, quality of life; RCT, randomized controlled trial; SIBO, small intestinal bacterial overgrowth; *Saccharomyces boulardii*, *Saccharomyces boulardii*.

### 3.3 Clinical response *versus* test-based outcomes

Based on a qualitative study-level classification, 27 of the 39 included studies reported both clinical and post-treatment test-based outcomes and could therefore be evaluated for concordance. Of these, 11 showed discordant or only partially concordant findings, 14 showed broadly concordant improvement in clinical and test-based outcomes, and two showed no significant benefit in either outcome.

Among studies with discordant findings, the most frequent pattern was symptomatic improvement despite limited breath-test normalization. This pattern was observed in studies involving diarrhea-predominant irritable bowel syndrome (IBS-D), postoperative colorectal cancer, and functional bloating, as well as in a study evaluating a multimodal treatment strategy (Redo et al., 2024; Jo et al., 2024; Zhuang et al., 2020; Deng et al., 2016).

A related divergence in treatment ranking was observed in the comparative three-antibiotic trial: metronidazole achieved the highest breath-test normalization rate, whereas rifaximin was associated with the greatest reductions in abdominal pain and bloating (Von Muhlenbrock et al., 2025).

By contrast, several studies reported closer alignment between test-based and clinical outcomes, with symptom improvement occurring predominantly among patients who achieved breath-test normalization. This pattern was observed in adults with IBS-associated SIBO, patients with systemic sclerosis, and children with IBS (Pimentel et al., 2000; Scarpellini et al., 2013; Yang et al., 2008; Parodi et al., 2008).

Two placebo-controlled trials found no significant between-group differences in neither clinical improvement, nor breath-test normalization following rifaximin treatment (Collins and Lin, 2011; Tuteja et al., 2019).

Concordance between clinical response and test-based outcomes was inconsistent across studies.

### 3.4 Combination, adjunctive, and comparative treatment strategies

Combination studies were interpreted according to their comparator structure: studies with an antibiotic-only arm allowed assessment of the incremental effect of the adjunctive intervention, whereas studies in which several treatment components differed between groups were summarized at the regimen level.

In patients with IBS and methane-positive breath tests, rifaximin plus neomycin produced the highest clinical response rate (85%, compared with 63% for neomycin and 56% for rifaximin) and significantly greater methane elimination (87% vs. 33% and 28%, respectively) (Low et al., 2010). Adding partially hydrolyzed guar gum to rifaximin significantly increased GBT normalization (from 62.1% to 85.0%) in the intention-to-treat analysis and to 87.1% in the per-protocol analysis, whereas symptomatic improvement among patients who achieved GBT normalization was similar between groups (Furnari et al., 2010). In systemic sclerosis-associated SIBO, metronidazole plus *S. boulardii* achieved the highest hydrogen breath-test normalization rate (55%, compared with 33% for *S. boulardii* and 25% for metronidazole alone), while symptom improvement was observed primarily in the *S. boulardii*-containing groups (García-Collinot et al., 2020). Neither mosapride nor trimebutine significantly increased breath-test normali-zation when added to rifaximin. The rifaximin–mosapride combination was associated with improvement in chest discomfort, whereas the rifaximin–trimebutine combination was associated with improvement in bloating (Kim et al., 2022; Jo et al., 2024). In the multimodal study, adjunctive interventions added to antibiotics and a low-FODMAP diet did not significantly improve breath-test gas outcomes, although normalization of clinical manifestations was more frequent in the intervention group among patients with methane-predominant SIBO. Because several adjunctive components differed between groups, the findings were attributed to the complete regimen (Redo et al., 2024). In patients with concomitant *Helicobacter pylori* infection, both amoxicillin-based triple regimens significantly reduced lactulose hydrogen breath test (LHBT) hydrogen levels and abdominal pain, with no significant between-group differences between the rifaximin and metronidazole-containing regimens (Konrad et al., 2018).

Strategies evaluated as alternatives rather than as concurrent combinations were considered separately. In patients with IBS and SIBO, both antibiotic and microbiota-directed treatments were associated with higher clinical response rates (89.7% and 91.7% vs. 38.5%) and breath-test negative conversion rates (76.9% and 86.1% vs. 23.1%) than placebo (Yu et al., 2021). In a small randomized open-label pilot trial, three-times-daily rifamycin SV MMX was associated with greater symptomatic improvement (66.7% vs. 25.0%) and a higher proportion of patients achieving both symptomatic and breath-test improvement (53.3% vs. 25.0%) than twice-daily dosing (Connor et al., 2025). In a retrospective cohort, rotating antibiotic regimens were associated with a higher rate of breath-test-defined remission than single-antibiotic regimens (70.0% vs. 50.8%, p = 0.050) (Richard et al., 2021). In a small randomized open-label pilot trial, metronidazole produced a greater reduction in flatus-incontinence episodes than simethicone plus activated charcoal but did not significantly improve other gastrointestinal symptoms or quality-of-life scores (Melchior et al., 2017).

### 3.5 Factors associated with treatment response

Response to rifaximin varied according to breath-test status and baseline gut microbial and inflammatory profiles. In patients with IBS-D, breath-test-positive patients showed improvement across a broader range of gastrointestinal outcomes, whereas improvement in the breath-test-negative subgroup was largely limited to abdominal pain. Breath-test-negative patients also had a distinct baseline profile, characterized by more pronounced systemic and colonic inflammatory signals and enrichment of potentially pathogenic or opportunistic taxa, including *Enterobacter, Enterococcus, Escherichia–Shigella, Klebsiella,* and *Cronobacter* (Liu et al., 2022).

Baseline fecal bacterial composition was also associated with the pattern of clinical response to rifaximin. In another study of patients with IBS-D, those whose baseline fecal bacterial profiles differed from those of healthy controls showed improvement across a broader range of symptoms than patients whose profiles were similar to those of healthy controls. Rifaximin also produced more pronounced changes in fecal bacterial structure and microbial interaction patterns in the subgroup with baseline fecal dysbiosis (Li et al., 2020).

Objective evidence of bacterial overgrowth and quantitative upper-gut bacterial burden were also associated with treatment response. Symptom resolution after norfloxacin was more frequent among patients with culture-confirmed SIBO than among those with bacterial counts below 10^3^ CFU/mL. In case of patients with respiratory tests below the conventional diagnostic threshold for SIBO, response also tended to be more frequent in those with bacterial counts of 10^3^ to <10^5^ CFU/mL than in those with counts below 10^3^ CFU/mL (Ghoshal et al., 2016).

Beyond patient-related factors, response also varied according to therapeutic strategy, with selected combination and multimodal regimens associated with improvement in specific clinical outcomes (García-Collinot et al., 2020; Redo et al., 2024; Kim et al., 2022; Jo et al., 2024). In the study providing 6-month follow-up, however, symptomatic benefit was most evident during the first 2 months and diminished progressively thereafter, indicating limited durability of response (Ghoshal et al., 2016).

Key determinants of treatment response and sources of variability across studies are summarized in Table 5.

**TABLE 5:** Reported factors associated with treatment response across included studies.

Author (Year)	Factor associated with treatment response	Reported finding	Study population/context
(Liu et al., 2022)	Breath-test-positive status	Greater symptomatic improvement after rifaximin was reported in breath-test-positive than in breath-test-negative patients	Adults with IBS-D stratified by breath test status
(Li et al., 2020)	Baseline gut microbiota composition	Baseline fecal microbial profiles were associated with clinical response to rifaximin	Adults with IBS-D
(Kim et al., 2022)	Rifaximin plus mosapride	Greater improvement in gas-related symptoms was reported with rifaximin-based therapy; breath-test normalization rates did not differ significantly among groups	Adults with functional dyspepsia and SIBO
(Jo et al., 2024)	Add-on trimebutine	Improvement in bloating was reported without a higher breath-test normalization rate	Adults with functional bloating and SIBO
(García-Collinot et al., 2020)	Antibiotic–probiotic combination	A higher breath-test normalization rate was reported with metronidazole plus Saccharomyces boulardii than with metronidazole alone	Adults with systemic sclerosis and SIBO
(Ghoshal et al., 2016)	Confirmed SIBO status	Symptom resolution after antibiotic therapy was reported more frequently in IBS patients with confirmed SIBO than in those without objective evidence of overgrowth	Adults with IBS stratified by SIBO status
(Richard et al., 2021)	Rotating antibiotic regimens	Higher breath-test-defined remission rates were reported with rotating regimens than with single-course antibiotic therapy	Adults with SIBO
(Redo et al., 2024)	Multimodal therapy	Greater clinical improvement was reported with multimodal treatment; breath-test gas outcomes did not differ significantly between groups	Adults with SIBO
(Scarpellini et al., 2013)	LBT normalization	Gastrointestinal symptom scores improved significantly among children whose LBT normalized, whereas persistent LBT positivity was not associated with significant VAS improvement	Children with IBS and SIBO
(Von Muhlenbrock et al., 2025)	Antibiotic regimen	Metronidazole showed the highest post-treatment breath-test negativity rate, whereas rifaximin was associated with greater symptom reduction and fewer adverse effects	Adults with IBS and SIBO

Abbreviations: IBS, irritable bowel syndrome; IBS-D, diarrhea-predominant irritable bowel syndrome; IL-1β, interleukin-1, beta; IL-10, interleukin-10; LBT, lactulose breath test; SIBO, small intestinal bacterial overgrowth; VAS, visual analogue scale.

## 4 Discussion

### 4.1 Heterogeneity of antibiotic efficacy

The heterogeneity observed across studies likely reflects differences in diagnostic criteria and breath-test methodology, as well as variation in patient populations, rifaximin regimens, and hydrogen-versus methane-associated breath-test phenotypes (Tuteja et al., 2019; Furnari et al., 2019; Kim et al., 2022). These findings suggest that antibiotic response may be greater when treatment is directed toward patients with objectively documented overgrowth, whereas symptom-based selection alone may be less reliable because gastrointestinal manifestations can overlap with those of the underlying disorder (Ghoshal et al., 2016; Furnari et al., 2019). For example, norfloxacin produced better short-term outcomes in IBS patients with conventionally defined SIBO than in those without confirmed SIBO, highlighting the potential importance of microbiologically informed patient selection (Ghoshal et al., 2016). Conversely, among Gulf War veterans with non-constipated IBS, rifaximin did not provide significant benefits over placebo in terms of gastrointestinal symptoms, quality of life, or LHBT normalization (Tuteja et al., 2019).

This overall pattern is broadly consistent with previous quantitative syntheses. In a meta-analysis of 32 studies including 1,331 adults, rifaximin was associated with pooled post-treatment test normalization rates of 70.8% by intention-to-treat and 72.9% per protocol, with adverse events reported in 4.6% of patients; however, between-study heterogeneity was substantial and the overall quality of the evidence was poor (Gatta and Scarpignato, 2017). A subsequent meta-analysis found that antibiotics increased symptomatic response compared with placebo or no treatment (RR 2.46, 95% CI 1.33–4.55), while IBS patients with test-positive SIBO responded more frequently than those without SIBO (51.2% vs. 23.4%; RR 2.07, 95% CI 1.40–3.08) (Takakura et al., 2024). A systematic review published after completion of our search reported similar rifaximin estimates and recurrence rates of approximately 40%–45% within 9–12 months, but rated most treatment and recurrence evidence as low certainty because of risk of bias, heterogeneity, and imprecision (Choudhary et al., 2026). Given that this review incorporated earlier meta-analyses and overlapping primary studies, its pooled estimates should be regarded as contextual corroboration rather than as an independent body of evidence.

Microbiome-based studies suggest that rifaximin response may vary according to baseline fecal microbial profiles and, in some cohorts, breath-test status; however, the predictive value of breath testing has not been consistent across studies, indicating that bacterial overgrowth or dysbiosis may not contribute equally to symptom generation across patients (Li et al., 2020; Liu et al., 2022; Tuteja et al., 2019).

Diagnostic uncertainty may provide a further explanation for inconsistent treatment effects. In a culture-based study of IBS, conventional SIBO, defined as >10^5^ CFU/mL of colonic bacteria, was detected in only 4% of both patients and healthy controls, whereas mildly increased small-bowel bacterial counts (>5 × 10^3^ CFU/mL) were significantly more frequent in IBS (43% vs. 12%) (Posserud et al., 2007). Similarly, differences in breath-test substrates, gas measurements, positivity thresholds, and operational definitions may alter SIBO classification and potentially lead to the inclusion of patients whose test abnormalities do not reflect clinically relevant small-bowel overgrowth (Collins and Lin, 2011; Enko et al., 2014; Enko et al., 2015).

Despite these limitations, specific breath-test characteristics may help identify clinically relevant subgroups. In selected IBS cohorts, lactulose breath-test curve morphology was associated with subsequent antibiotic response, whereas methane excretion was linked to constipation-predominant symptoms; symptom improvement was also greatest when antibiotic treatment resulted in lactulose breath test (LBT) normalization (Kasir et al., 2016; Pimentel et al., 2003). Phenotype-dependent variation has also been reported outside IBS. In cirrhosis, hydrogen-producing SIBO was independently associated with greater liver dysfunction and was more frequent among patients with covert hepatic encephalopathy, whereas methane-positive overgrowth, classified as IMO, showed no comparable associations (Yokoyama et al., 2022).

Underlying predisposing mechanisms may also be relevant to the persistence and recurrence of SIBO. In a large retrospective cohort, impaired intestinal clearance, drug-induced immunosuppression, and levothyroxine therapy were associated with SIBO, underscoring the importance of interpreting antibiotic outcomes within the broader pathophysiological context (Brechmann et al., 2017).

### 4.2 Clinical response *versus* test-based outcomes

As summarized in Section 3.3, clinical response and post-treatment test-based outcomes did not consistently align. The most frequent pattern was clinical improvement despite incomplete or modest breath-test normalization, whereas test normalization was not invariably accompanied by corresponding clinical benefit. Clinical response, breath-test normalization, and microbiological eradication should therefore be considered distinct treatment outcomes.

One possible explanation for this dissociation is that symptom relief reflects reduced bacterial fermentation or altered microbial metabolic activity rather than complete bacterial clearance alone. Fermentation products may contribute to bloating, distension, abdominal discomfort, and diarrhea, yet symptom occurrence does not consistently parallel breath-hydrogen findings (Simr et al., 2006; Goebel-Stengel et al., 2014). Antibiotics may therefore modify bacterial fermentation and carbohydrate handling in ways that reduce symptoms before complete breath-test normalization is achieved, although the relationship between these effects remains uncertain (Nucera et al., 2005; Barrett et al., 2009). Consistent with this interpretation, partial suppression of microbial gas production may be sufficient for symptom relief even when breath-test normalization remains incomplete (Kim et al., 2022; Jo et al., 2024).

Rifaximin may also exert effects beyond direct bacterial eradication. An experimental study showed that it altered epithelial-cell expression of proteins involved in cytoskeletal organization, transcription and translation, and cellular metabolism, suggesting a potential cytoprotective effect (Schrodt et al., 2013). Microbiome and metabolomic studies further suggest that rifaximin can influence microbial function, with changes in short-chain fatty acids, other bacterial metabolites, and microbiota–metabolite interactions despite only modest alterations in overall microbiota composition (Bajaj et al., 2013; Maccaferri et al., 2010). Although these studies did not assess breath-test outcomes, such functional modulation provides a plausible mechanistic explanation for clinical improvement without complete test normalization.

Host-related mechanisms may provide an additional explanation for the divergence between clinical and breath-test outcomes, particularly through microbiome–gut–brain interactions involving tryptophan metabolism (Chojnacki et al., 2022). Reported improvements in anxiety and depression after rifaximin therapy, accompanied by changes in tryptophan metabolism, suggest that clinical response may involve host–microbiome pathways beyond direct suppression of bacterial overgrowth (Chojnacki et al., 2022). This interpretation is also consistent with evidence that some breath-test abnormalities have limited disease specificity and may persist independently of clinically relevant symptoms (Barrett et al., 2009).

Evidence from functional gastrointestinal disorders further suggests that rifaximin may relieve symptoms even when treatment is not restricted to patients with breath-test-confirmed SIBO. In IBS, rifaximin improved global symptoms and bloating compared with placebo; however, breath-test status was either not reported or was not used to select participants in the principal trials (Pimentel et al., 2006; Ford et al., 2014). In functional dyspepsia, rifaximin improved global dyspeptic symptoms and postprandial fullness/bloating in patients with a negative baseline LHBT (Tan et al., 2017). These findings are consistent with the possibility that symptom improvement may not depend on the eradication of breath-test-defined overgrowth, although the underlying mechanisms remain uncertain.

These discrepancies have methodological and clinical implications. Trials that rely on breath-test normalization as the primary endpoint may not fully capture clinical benefit, whereas symptom-based outcomes alone may overlook relevant changes in breath-gas production or microbial composition (Liu et al., 2022; Kim et al., 2022; Jo et al., 2024). Future studies should therefore report clinical response and breath-test normalization as separate outcomes and, where microbiological assessment is feasible, report microbiological eradication independently.

### 4.3 Combination, adjunctive, and comparative treatment strategies

Combination studies were interpreted according to their comparator structure, with outcomes attributed to the complete regimen whenever the independent contribution of the antibiotic component could not be isolated.

Combination therapy may be beneficial in selected clinical settings, particularly in methane-positive phenotypes. In a small randomized trial involving patients with methane-positive constipation-predominant IBS, rifaximin combined with neomycin produced greater improvements in constipation, straining, and bloating than neomycin alone, while post-treatment methane levels ≤3 ppm were associated with less severe constipation (Pimentel et al., 2014). In a small uncontrolled pediatric study conducted in a socioeconomically disadvantaged community in Osasco, Brazil, asymptomatic schoolchildren aged 6–10 years with SIBO defined by lactulose breath testing received trimethoprim–sulfamethoxazole plus metronidazole. One month after treatment, 19 of 20 children had a negative breath test, although methane production did not decrease significantly (Tahan et al., 2013). Considered alongside the findings in methane-positive constipation-predominant IBS, these results suggest that combination regimens may exert differential effects on hydrogen-producing bacteria and methanogenic communities.

Treatment strategy may itself contribute to outcome variability. In one study, adding herbal supplements, probiotics, prebiotics, and glutamine to antibiotics and a low-FODMAP diet was associated with higher clinical remission among patients with methane-positive/IMO phenotype, although it did not improve breath-gas normalization (Redo et al., 2024). Together with evidence that treatment response varies across distinct microbial and inflammatory phenotypes, these findings support further evaluation of individualized multimodal strategies in selected patients (Redo et al., 2024; Liu et al., 2022). Retrospective comparative data have also associated rotating antibiotic regimens with higher breath-test-defined remission rates than single-antibiotic regimens (Richard et al., 2021). Collectively, these observations suggest that regimen composition—including antibiotic rotation and the addition of probiotics or other adjunctive interventions to antibiotic and dietary therapy—may contribute to between-study variability in breath-test-defined remission and symptom control (García-Collinot et al., 2020; Redo et al., 2024; Richard et al., 2021).

Nevertheless, the benefits of combination therapy have not been consistent across clinical and test-based outcomes. In one randomized trial, adding the motility-modulating agent trimebutine to rifaximin improved bloating but did not increase complete GBT normalization compared with rifaximin alone (34.1% vs. 35.9%) (Jo et al., 2024). More broadly, rifaximin response has also varied according to breath-test status and baseline microbial phenotype (Liu et al., 2022). Moreover, multimodal regimens may make it difficult to determine which treatment component accounts for the observed benefit and may therefore complicate comparisons across studies (García-Collinot et al., 2020; Redo et al., 2024). Baseline differences in microbial composition may further contribute to heterogeneity in rifaximin response (Li et al., 2020).

A 2025 network meta-analysis of 30 randomized trials suggested that relative treatment rankings may vary according to clinical context. Among patients with concomitant functional gastrointestinal disorders, rifaximin combined with a gastrointestinal motility agent ranked first, although rifaximin alone also ranked highly (Zhang et al., 2025). However, the analysis grouped heterogeneous interventions and applied a broad, non-uniform definition of treatment success that encompassed bacterial-count reduction, breath-test normalization, or clinical improvement. Its rankings should therefore be regarded as hypothesis-generating rather than definitive.

Comparative antibiotic studies further indicate that the preferred regimen may depend on the outcome being prioritized. In the study by von Muhlenbrock et al., the antibiotic associated with the highest breath-test normalization rate differed from the regimen showing the most favorable balance of symptom improvement and tolerability (Von Muhlenbrock et al., 2025).

Combination therapy may provide additional symptom control in selected clinical settings, although its benefits have not been consistent across populations or treatment outcomes. These findings, together with evidence that rifaximin response differs according to breath-test and microbial phenotype, indicate that each strategy should be interpreted in light of the comparator used, the patient phenotype, and the relative weight assigned to clinical response, test-based outcomes, and tolerability (Redo et al., 2024; Liu et al., 2022; Kim et al., 2022; Jo et al., 2024).

### 4.4 Factors associated with treatment response

Treatment response in SIBO may depend not only on antibiotic choice but also on whether bacterial overgrowth is objectively confirmed and contributes to the presenting symptoms. Among patients with IBS, symptom response to norfloxacin was greater in those with culture-confirmed SIBO than in those without confirmed overgrowth (Ghoshal et al., 2016). Similarly, patients with IBS-D who were breath-test positive showed improvement across a broader range of symptoms after rifaximin than those who were breath-test negative (Liu et al., 2022). Conversely, rifaximin was not superior to placebo in Gulf War veterans with non-constipated IBS, in whom treatment was not restricted to patients with a positive breath test (Tuteja et al., 2019).

Baseline microbial characteristics may further help explain variation in treatment response. In patients with IBS-D, pretreatment fecal bacterial composition was associated with the response to rifaximin, with broader symptomatic improvement among patients whose microbial profiles differed from those of healthy controls (Li et al., 2020). Together, the distinct microbial and inflammatory profiles observed in breath-test-positive and breath-test-negative patients with IBS-D, along with their differential response to rifaximin, suggest that breath-test status may help distinguish clinically relevant subgroups, although the evidence remains exploratory (Liu et al., 2022).

Diagnostic methodology may also affect the apparent test-based response to treatment. Differences in breath-test substrate, sampling intervals, and diagnostic thresholds may in-fluence both patient classification and the interpretation of post-treatment breath-test results (Saad and Chey; Simr et al., 2006). Variation in treatment outcomes may also reflect the underlying clinical context, as studies in disease-specific populations—including systemic sclerosis, cystic fibrosis, and concomitant *H. pylori* infection—have reported differing patterns of breath-test normalization and symptom improvement (García-Collinot et al., 2020; Furnari et al., 2019; Konrad et al., 2018).

Treatment regimen represents another potential source of variation. In a randomized open-label pilot study of rifamycin SV MMX, both dosing regimens were associated with symptom improvement and breath-test normalization, with greater symptomatic improvement observed with three-times-daily dosing (Connor et al., 2025). Given the small sample size and exploratory design, these findings require confirmation in adequately powered comparative trials.

The available evidence points toward a stratified rather than uniform therapeutic approach that considers diagnostic certainty, baseline microbial characteristics, clinical context, and treatment regimen. Most proposed predictors or modifiers of response, however, remain preliminary and require prospective validation.

### 4.5 Recurrence, antimicrobial stewardship, and future directions

Recurrence remains an important limitation of antibiotic-based treatment. In one prospective follow-up study, GBT positivity recurred after successful normalization with rifaximin in 12.5% of patients at 3 months, 27.5% at 6 months, and 43.7% at 9 months. Recurrence was also associated with relapse of gastrointestinal symptoms. Older age, chronic proton pump inhibitor use, and a history of appendectomy were independently associated with recurrence (Lauritano et al., 2008). A systematic review published after completion of our search placed these findings in a broader context, reporting recurrence rates of approximately 40%–45% within 9–12 months after successful eradication. However, the certainty of this evidence was low because the estimates were based on only three small, largely observational cohorts with heterogeneous follow-up intervals and maintenance strategies (Choudhary et al., 2026).

Persistent dysmotility, anatomical abnormalities, and continued exposure to predisposing medications may promote recurrence when these factors remain uncorrected. They should therefore be reassessed and, where possible, addressed before repeated antibiotic therapy is considered (Ghoshal et al., 2022; Silva et al., 2025; Quigley et al., 2020).

Evidence to guide retreatment remains limited. Repeated courses of rifaximin have been used after symptom recurrence, whereas rotating antibiotic regimens have been evaluated more broadly as a treatment strategy for SIBO. However, these retrospective studies do not establish the optimal timing, duration, or regimen for retreatment, nor whether any approach reduces subsequent recurrence (Richard et al., 2021; Yang et al., 2008).

These uncertainties underscore the importance of antimicrobial stewardship, as treatment remains largely empiric and antibiotic selection, dosing, and duration are not well standardized (Ghoshal et al., 2022; Quigley et al., 2020). Rifaximin is poorly absorbed systemically, and a previous metaanalysis reported a pooled adverse-event rate of 4.6% (95% CI: 2.3%–7.5%), although the underlying evidence was heterogeneous (Gatta and Scarpignato, 2017). Nevertheless, potential risks of repeated or prolonged antibiotic use include selection for resistant organisms, unintended alterations of the intestinal microbiota, treatment-related adverse events, and *Clostridioides difficile* infection (Silva et al., 2025; Quigley et al., 2020).

Where feasible, objective diagnostic confirmation before initial treatment, correction of underlying causes, use of a time-limited treatment course, and reassessment of recurrent symptoms before retreatment may help minimize unnecessary antibiotic exposure (Silva et al., 2025; Quigley et al., 2020). Repeated or prolonged regimens should therefore be reserved for selected patients with persistent or recurrent disease, given the limited evidence supporting long-term antibiotic strategies.

Although non-antibiotic antimicrobial therapies were outside the predefined scope of this review, they warrant consideration in the context of repeated antibiotic exposure and antimicrobial stewardship. In a retrospective, non-randomized cohort, Chedid et al. reported negative follow-up lactulose breath tests in 46% of patients treated with multi-herbal formulations and 34% of those receiving rifaximin, with no significant difference between groups (p = 0.24) (Chedid et al., 2014). More recently, an open-label non-inferiority randomized trial, currently available as a conference abstract, found berberine to be non-inferior to rifaximin for breath-test conversion and secondary clinical outcomes at both two and 6 weeks (Gao et al., 2025). Although these findings suggest that non-antibiotic antimicrobial strategies may have therapeutic potential, further adequately powered, high-quality randomized controlled trials are needed to confirm the efficacy, safety, and long-term outcomes of herbal antimicrobial therapies in patients with SIBO before these approaches can be recommended in routine clinical practice.

Future studies should use standardized diagnostic definitions and predefined treatment protocols, with clinical response reported separately from breath-test normalization, microbiological eradication, and methane/IMO-related outcomes. Follow-up should be sufficiently long to document recurrence and retreatment, while also capturing cumulative antibiotic exposure, adverse events, microbiome-related effects, and the potential emergence of antimicrobial resistance.

In pediatric practice, evidence guiding antibiotic selection, dosing, and treatment duration remains particularly limited, and currently suggested regimens are based largely on expert opinion because comparative pediatric data are scarce (Puoti et al., 2025). More broadly, pediatric *H. pylori* studies also suggest that antibiotic efficacy and tolerability may differ across regimens. In a Romanian cohort, a metronidazole-based triple regimen achieved higher eradication rates and fewer adverse effects than a clarithromycin-based regimen (Rosu et al., 2022). Future pediatric research should therefore compare age-specific regimens, including antibiotic choice, dose, and duration; use validated symptom and quality-of-life measures; incorporate predefined safety and antimicrobial-resistance monitoring; and provide sufficiently long follow-up to assess recurrence.

### 4.6 Strengths and limitations

A major strength of this review is its broad evaluation of antibiotic-based treatment across adult and pediatric populations, different clinical settings, and studies published between 1 January 2000 and 2 April 2026. Clinical response, breath-test normalization, microbiological eradication, methane-related outcomes, recurrence, treatment strategies, and factors associated with treatment response were considered separately. The distinction between bacterial SIBO and methane-positive/IMO phenotypes, together with the use of design-specific risk-of-bias tools, further strengthened the interpretation of the available evidence.

Several limitations should be acknowledged. Considerable clinical and methodological heterogeneity was present across study populations, diagnostic substrates and thresholds, antibiotic regimens, comparator groups, follow-up intervals, and outcome definitions. These differences limited direct comparisons between interventions and precluded a meaningful quantitative synthesis. In addition, several studies were small, uncontrolled, retrospective, or at moderate to serious risk of bias, and validated symptom instruments were not used consistently.

Combination and multimodal studies frequently did not allow the independent contribution of the antibiotic component to be isolated. Participant-level associations between clinical response and breath-test normalization were also inconsistently reported, while recurrence, retreatment, and longer-term safety were assessed in only a minority of studies. Historical definitions of methane positivity varied substantially, limiting direct comparison with current IMO criteria.

Pediatric evidence remained sparse and heterogeneous, with limited information regarding age-specific dosing, validated patient-reported outcomes, safety monitoring, recurrence, and longer-term treatment effects. Restriction to English-language full-text publications may also have introduced language and availability bias. Accordingly, the findings provide a structured qualitative overview of the available evidence but should not be interpreted as establishing comparative superiority between regimens or a definitive treatment algorithm.

## 5 Conclusion

The available evidence does not identify a universally superior antibiotic across all SIBO phenotypes. In adults with bacterial SIBO, rifaximin appears to be the best-supported option overall because it is the most extensively studied agent and generally offers the most favorable balance between symptom improvement and tolerability. However, metronidazole achieved the highest breath-test normalization rate in the only direct three-antibiotic comparison, indicating that the preferred treatment may differ according to whether clinical improvement or test normalization is prioritized. In methane-positive/IMO phenotypes, rifaximin combined with neomycin produced better clinical response and methane elimination than either agent alone, although this evidence remains based on small studies.

In children, the available evidence is insufficient to establish a preferred antibiotic. Rifaximin outcomes were inconsistent, and the high breath-test response reported with trimethoprim–sulfamethoxazole plus metronidazole was derived from a small uncontrolled cohort. Antibiotic selection should therefore be individualized according to diagnostic confidence, gas phenotype, underlying predisposing condition, safety considerations, and recurrence risk. Treatment success should incorporate symptom improvement, objective response, tolerability, and recurrence rather than breath-test normalization alone.

## Data Availability

The original contributions presented in the study are included in the article/Supplementary Material, further inquiries can be directed to the corresponding author.
