# Supplementary material for: Antibiotic therapy for small intestinal bacterial overgrowth: a systematic review of comparative efficacy, clinical–test discordance, and pediatric evidence gaps
Source: Front Pharmacol. 2026 Sep 10;17:1908516. doi: 10.3389/fphar.2026.1908516 (PMC13601715; doi:10.3389/fphar.2026.1908516)
Supplement: Supplementary file 1 [file Table1.docx]

# Supplementary Material

**Table S1.** Detailed characteristics of the included studies evaluating antibiotic-based therapy for SIBO and methane-positive/IMO phenotypes

| Study | Design | Population | N | Diagnostic test | Antibiotic regimen | Comparator | Follow-up | Microbiological outcome | Clinical outcome |
| --- | --- | --- | --- | --- | --- | --- | --- | --- | --- |
| **Di Stefano (2000)** (93) | Double-blind RCT | Adults with SIBO | 21 | GHBT | Rifaximin 1200 mg/day for 7 days | Chlortetracycline 1 g/day for 7 days | 3 days post-treatment | Breath-test normalization: 70% vs 27% | Greater symptom improvement with rifaximin |
| **Lauritano (2005)** (94) | Randomized dose-finding study | Adults with SIBO | 90 | GHBT | Rifaximin 600, 800, or 1200 mg/day for 7 days | Dose comparison | 1 month post-therapy | GBT normalization: 17%, 27%, and 60% | Highest normalization with 1200 mg/day; tolerability preserved |
| **Majewski (2007)** (95) | Prospective open-label study | Symptomatic adults with SIBO | 20 | H₂/CH₄ GHBT | Rifaximin 800 mg/day for 4 weeks | None | Within 7 days post-therapy | GBT normalized in approximately half | Symptoms improved, especially in diarrhea-predominant presentations |
| **Scarpellini (2007)** (96) | Prospective randomized study | Adults with SIBO | 80 | H₂/CH₄ GHBT | Rifaximin 1600 vs 1200 mg/day for 7 days | Dose comparison | 1 month post-therapy | Normalization: 80% vs 58% in the ITT analysis | Higher-dose regimen was more effective, with similar tolerability |
| **Tuteja (2019)** (49) | Double-blind placebo-controlled RCT | Adults with IBS | 50 | LHBT | Rifaximin | Placebo | Post-treatment | No significant breath-test benefit | No significant symptom benefit vs placebo |
| **Lee (2019)** (97) | Retrospective observational study | Adults with IBS/SIBO | 153 | LHBT | Rifaximin monotherapy | None | Post-treatment | NR | Symptoms improved without meaningful weight or metabolic changes |
| **Zhuang (2020)** (58) | Prospective study | Adults with IBS-D | 78 | LHBT | Rifaximin 400 mg twice daily for 2 weeks | None | Post-treatment reassessment | Partial LHBT normalization | GI symptoms and quality of life improved |
| **Li (2020)** (44) | Prospective mechanistic study | Adults with IBS-D | 89 | GHBT | Rifaximin for 2 weeks | None | Post-treatment microbiota assessment | Eradication was not the primary endpoint | Clinical response was associated with baseline gut microbiota composition |
| **Chojnacki (2022)** (82) | Before–after interventional study | Adults with SIBO-D/SIBO-C | 120 | LHBT | Rifaximin 1200 mg/day; repeated treatment cycles | None | After treatment cycles | Eradication was not the primary endpoint | GI and psychological symptoms improved |
| **Liu (2022)** (45) | Clinical/mechanistic study | Adults with IBS-D stratified by SIBO status | 60 | Breath test | Rifaximin | SIBO-positive vs SIBO-negative response comparison | Post-treatment | Eradication was not the primary endpoint | Greater symptom improvement in SIBO-positive patients |
| **García-Cedillo (2025)** (98) | Prospective open-label pilot study | IBS without constipation, SIBO, and lactose intolerance | 25 | LHBT and lactose HBT | Rifaximin-alpha 400 mg every 8 hours for 2 weeks | None | 4 weeks post-intervention | Negative SIBO LHBT in 32%; lactose maldigestion decreased | Symptoms, lactose maldigestion, and lactase-related measures improved |
| **Pimentel (2000)** (25) | Prospective database/open-label treatment study | IBS with abnormal LHBT suggestive of SIBO | 202 with IBS; 47 with follow-up | LHBT | Open-label antibiotics | Successfully eradicated vs non-eradicated patients | Follow-up breath test | Eradication achieved in 25/47 patients | Eradication was associated with IBS symptom improvement; 48% no longer met Rome criteria |
| **Madrid (2001)** (99) | Randomized controlled study | Adults with liver cirrhosis | 34 | H₂ breath test | Alternating norfloxacin and neomycin for 6 months | Cisapride or placebo | 3 and 6 months | SIBO decreased with antibiotics | Motility, OCTT, SIBO, and liver-function indices improved |
| **Esposito (2007)** (23) | Observational study | Adults initially diagnosed with IBS | 73; 33 SIBO-positive | Breath test | Rifaximin 1200 mg/day for 7 days; ciprofloxacin for persistent positivity | None | 1 week after rifaximin | 59.4% became breath-test negative after rifaximin; ciprofloxacin provided limited additional benefit | Symptoms decreased significantly after rifaximin |
| **Yang (2008)** (60) | Retrospective chart review | IBS with abnormal LBT | 98 | LBT | Rifaximin, neomycin, or other antibiotics; retreatment courses | Antibiotic comparison | Variable clinical follow-up | LBT normalization predicted response | Rifaximin showed higher clinical response and better retreatment success |
| **Lauritano (2008)** (87) | Prospective follow-up study | Adults successfully decontaminated after rifaximin therapy | 80 | GHBT | Initial rifaximin 1200 mg/day for 7 days, followed by observation | None | 3, 6, and 9 months | Recurrence: 12.5%, 27.5%, and 43.7%, respectively | Recurrence was associated with symptom relapse; older age, appendectomy, and PPI use predicted recurrence |
| **Ghoshal (2016)** (30) | Double-blind placebo-controlled RCT | Adults with IBS stratified by SIBO status | 80 | GHBT and culture | Norfloxacin for 10 days | Placebo | 6 months | Test normalization: 50% vs 0% | Symptom resolution was more frequent in SIBO-positive patients with IBS |
| **Melchior (2017)** (66) | Pilot randomized trial | Adults with SIBO and gas-related symptoms | 16 | GHBT | Metronidazole | Simethicone plus activated charcoal | Symptom-diary follow-up | NA | Metronidazole reduced gas-related symptoms |
| **Richard (2021)** (54) | Retrospective comparative cohort | Adults with SIBO | 223 | GHBT | Rotating antibiotics | Single-course antibiotics | Post-treatment breath test | Remission: 70.0% vs 50.8% | Rotating regimens were associated with better remission and QoL- and bloating-related outcomes |
| **Connor (2025)** (65) | Open-label pilot randomized trial | Adults with SIBO | 31 | Breath test | Rifamycin SV-MMX BID vs TID for 14 days | Dose-frequency comparison | Post-treatment | Breath-test response was reported and was higher with TID dosing | Higher-frequency dosing produced greater symptom improvement |
| **von Muhlenbrock (2025)** (53) | Prospective randomized double-blind comparative study | Adults with IBS and SIBO | 97 enrolled; 79 completed | H₂/CH₄ LHBT | Rifaximin vs ciprofloxacin vs metronidazole | Three-arm antibiotic comparison | 15 days post-treatment | Eradication: 59%, approximately 53–54%, and 79%, respectively | Metronidazole had the highest eradication rate; rifaximin produced greater improvement in pain and bloating and had better tolerability |
| **Low (2010)** (41) | Retrospective chart review | Methane-positive IBS/methane-positive or IMO phenotype | 74 | Methane-positive LBT | Rifaximin, neomycin, or rifaximin plus neomycin for 10 days | Antibiotic comparison | Follow-up clinical evaluation and breath test | Methane eradication: 87% with combination therapy vs 33% with neomycin vs 28% with rifaximin | Combination therapy produced the highest clinical response |
| **Furnari (2010)** (62) | Randomized clinical trial | Adults with SIBO and predisposing conditions | 77 | GHBT | Rifaximin 1200 mg/day with or without partially hydrolyzed guar gum for 10 days | Rifaximin alone | 1 month post-therapy | Eradication: 85–87.1% with combination therapy vs 62.1% with rifaximin alone | Clinical improvement was similar among patients with successful eradication |
| **García-Collinot (2020)** (33) | Open pilot randomized clinical trial | Systemic sclerosis with SIBO | 40 | Breath test | Metronidazole with or without *Saccharomyces boulardii* | Monotherapy/probiotic comparison | Post-treatment | Eradication: 55% with combination therapy vs 25% with metronidazole | Abdominal pain, bloating, and gas/flatulence improved |
| **Yu (2021)** (64) | Comparative clinical study | Adults with IBS/SIBO | 127 | Breath test | Multiple antibiotics | Microbiota-directed therapy or placebo comparison | Post-treatment | NR | Symptoms improved together with lower IL-1β and higher IL-10 levels |
| **Kim (2022)** (51) | Randomized blinded three-arm trial | Functional dyspepsia with SIBO | 83 | GHBT | Rifaximin, mosapride, or combination therapy for 2 weeks | Three-arm comparison | Post-treatment | Eradication: 32.1%, 17.2%, and 34.6%, respectively | Gas-related symptoms improved more with rifaximin-based therapy |
| **Jo (2024)** (52) | Double-blind placebo-controlled RCT | Functional bloating with SIBO | 80 | GHBT | Rifaximin plus trimebutine | Rifaximin plus placebo | Post-treatment | Eradication: 35.9% vs 34.1% | Bloating and gas-related symptoms improved without greater eradication |
| **Redondo-Cuevas (2024)** (34) | Prospective comparative interventional study | Adults with SIBO | 179 | H₂/CH₄ breath test | Antibiotics and low-FODMAP diet with or without adjunctive interventions | Standard vs multimodal approach | Post-treatment | Gas normalization did not differ significantly | Multimodal therapy improved symptoms, particularly in methane-predominant patients |
| **Jahraus (2007)** (55) | Retrospective case review | Patients undergoing abdominopelvic radiotherapy who developed GI symptoms suggestive of SIBO | 30 tested; 18 treated; 16 evaluable for clinical response | Glucose or lactulose hydrogen breath test | Rifaximin 400 mg BID or TID, corresponding to 800–1200 mg/day, throughout radiotherapy and for 2–4 weeks afterward | None | During radiotherapy and for 2–4 weeks following its completion; clinical assessment was not standardized | Seventeen of 30 patients had a positive baseline breath test; post-treatment breath-test normalization was not systematically assessed | Among 16 evaluable treated patients, 15/16 reported improvement in at least some presenting GI symptoms and 13/16 reported improvement in all presenting symptoms |
| **Parodi (2008)** (61) | Prospective clinical study | Systemic sclerosis with SIBO | 55 with systemic sclerosis; 30 SIBO-positive | LBT | Rifaximin 1200 mg/day for 10 days | Healthy controls for prevalence assessment | 1 month post-therapy | Eradication: 73.3% | Intestinal symptoms improved after eradication |
| **Franco (2015)** (56) | Retrospective cohort study | Patients undergoing outpatient upper endoscopy with quantitative duodenal aspirate culture | 1263 with aspirates; 384 culture-positive; 259 culture-positive patients treated | Quantitative aerobic culture of aspirates obtained from the third or fourth portion of the duodenum; positive result defined as >10⁵ CFU/mL | Antibiotic treatment, predominantly rifaximin; ciprofloxacin and metronidazole were also used; regimens and duration were not standardized | Antibiotic-treated vs untreated culture-positive patients | Mean clinical follow-up of 126 days, range 6–555 days | Culture-defined SIBO was identified in 384/1263 patients; post-treatment culture or microbiological eradication was not systematically assessed | Among culture-positive patients, clinical improvement did not differ significantly between treated and untreated patients: 53.0% vs 46.5% |
| **Deng (2016)** (59) | Prospective interventional study | Postoperative colorectal cancer patients with SIBO | 43 with colorectal cancer; 18 SIBO-positive | GHBT | Rifaximin for 10 days | Healthy controls for prevalence assessment | Post-treatment | 33.3% converted to GHBT-negative status | Overall GI symptoms improved, especially diarrhea |
| **Konrad (2018)** (63) | Comparative non-randomized study | SIBO with *H. pylori* infection | 62 | LHBT and ¹³C-UBT | Rifaximin plus amoxicillin | Standard regimen | Post-treatment | LHBT improvement reported | Symptoms improved in patients with SIBO/*H. pylori* co-infection |
| **Furnari (2019)** (50) | Open-label randomized controlled study | Cystic fibrosis with SIBO | 23 | GHBT | Rifaximin | No treatment | Post-treatment | Eradication: 90% vs 33.3% | Selected GI symptoms and nutritional parameters improved |
| **Chaudhry (2025)** (100) | Retrospective clinical utility study | Gas-bloat symptoms after antireflux surgery | 71; 40 SIBO-positive | H₂/CH₄ breath test | Antibiotics for SIBO-positive patients | SIBO-negative comparison | Post-treatment reassessment | Eradication was not the primary endpoint | Severe gas-bloat decreased; GERD-HRQL scores and patient satisfaction improved |
| **Chidambaram (2025)** (101) | Retrospective cohort study | Reflux or LPR symptoms with SIBO or IMO | 23 | H₂/CH₄ breath test | Antibiotics for SIBO/IMO | None | Post-treatment symptom scores | Eradication was not the primary endpoint | GERD-HRQL and RSI scores improved; PPI use decreased and surgery was avoided |
| **Collins (2011)** (4) | Double-blind placebo-controlled RCT | Children with chronic abdominal pain | 75 | LHBT | Rifaximin 550 mg TID for 10 days | Placebo | Post-treatment | LHBT normalization: 20% vs 14% | No significant symptom improvement vs placebo |
| **Lisowska (2011)** (102) | Interventional clinical study | Cystic fibrosis patients with SIBO | 26 | H₂/CH₄ breath test | Oral ciprofloxacin or intravenous ceftazidime plus amikacin | Route/subgroup comparison | After antibiotic therapy | Eradication was not the primary endpoint | Fat digestion and absorption improved, especially in the oral-antibiotic subgroup |
| **Scarpellini (2013)** (57) | Preliminary prospective open-label study | Children with IBS and SIBO | 50 enrolled; 33 treated | H₂/CH₄ LHBT | Rifaximin 600 mg/day for 7 days | None | Post-treatment | LHBT normalization: 64% | Abdominal pain, bloating, and flatulence improved mainly after successful normalization |

**Abbreviations:** ¹³C-UBT, carbon-13 urea breath test; BID, twice daily; CFU, colony-forming units; GBT, glucose breath test; GERD-HRQL, Gastroesophageal Reflux Disease Health-Related Quality of Life questionnaire; GHBT, glucose hydrogen breath test; GI, gastrointestinal; HBT, hydrogen breath test; IBS, irritable bowel syndrome; IBS-D, diarrhea-predominant irritable bowel syndrome; IMO, intestinal methanogen overgrowth; ITT, intention-to-treat; LBT, lactulose breath test; LHBT, lactulose hydrogen breath test; LPR, laryngopharyngeal reflux; MMX, multi-matrix system; NA, not applicable; NR, not reported; OCTT, orocecal transit time; PPI, proton pump inhibitor; QoL, quality of life; RCT, randomized controlled trial; RSI, Reflux Symptom Index; SIBO, small intestinal bacterial overgrowth; SIBO-C, constipation-predominant SIBO; SIBO-D, diarrhea-predominant SIBO; TID, three times daily.
